# Antibody-enabled structural biology and AI-driven antibody design

**DOI:** 10.3389/fphar.2026.1773629

**Published:** 2026-02-27

**Authors:** Khuram U. Ashraf, Satchal K. Erramilli

**Affiliations:** 1 Independent Researcher, Framingham, MA, United States; 2 Independent Researcher, Rockville, MD, United States

**Keywords:** antigen-binding fragments (fabs), artificial intelligence, cryo-EM, designed ankyrin repeat proteins (DARPins), GPCR, membrane protein, nanobodies, structural biology

## Abstract

Membrane proteins govern essential cellular processes, including ion transport, signal transduction, and molecular recognition, and collectively represent more than half of all current therapeutic targets. Yet their structural characterization remains challenging due to intrinsic instability, amphipathic surfaces, and conformational heterogeneity. Over the past decade, antibody-based approaches, spanning full-length immunoglobulins, antigen-binding fragments (Fabs), nanobodies, and engineered scaffolds such as designed ankyrin repeat proteins (DARPins), have transformed structural biology by stabilizing dynamic states, augmenting molecular weight for cryo-electron microscopy (cryo-EM), and enabling visualization of previously inaccessible complexes. In parallel, advances in artificial intelligence and machine learning have begun to enhance predictive modeling, accelerate structure determination, and guide rational design of protein-ligand and antibody–antigen interactions. This review examines how antibody engineering and AI-driven computation together are reshaping the landscape of structural biology and therapeutic discovery.

## 1 Introduction

Membrane proteins are central to cellular physiology, mediating ion transport, signal transduction, nutrient uptake, and molecular communication across biological membranes. Their amphipathic composition, conformational dynamics, and requirement for lipidic surroundings historically hindered structural determination (Carpenter et al., 2008). Early crystallographic and NMR studies relied heavily on detergents, engineered truncations, or thermostabilizing mutations, often at the expense of native activity, and still frequently yielded poorly ordered crystals or heterogeneous conformations (Bill et al., 2011).

**FIGURE 1:**
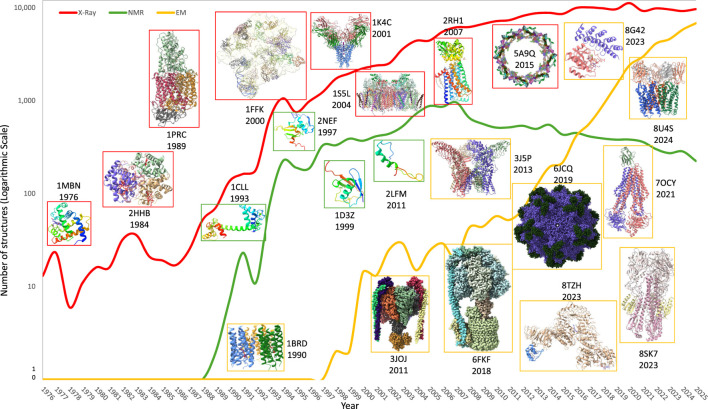
Evolution of structural biology methods and expanding access to complex biomolecular architectures. The number of experimentally determined macromolecular structures deposited per year in the Protein Data Bank (PDB) is shown for X-ray crystallography (red), nuclear magnetic resonance (NMR) spectroscopy (green), and electron microscopy (EM; gold), plotted on a logarithmic scale. Counts were derived from PDB deposition metadata based on the primary experimental method assigned at the time of structure release. Representative-landmark structures are highlighted to illustrate major methodological milestones and the progressive increase in structural complexity accessible to each technique, including early globular proteins, membrane proteins, large macromolecular assemblies, and dynamic multi-component complexes. The pronounced rise in EM-derived structures over the past decade reflects advances in detector technology, image processing algorithms, and sample-stabilization strategies, including the use of antibody fragments and engineered scaffolds. Together, these trends illustrate how methodological innovation, rather than biological feasibility alone, has driven the expanding reach of structural biology toward increasingly challenging and therapeutically relevant targets.

The past 15 years have transformed this landscape. Cryo-electron microscopy (cryo-EM), empowered by direct-electron detectors, improved image processing, and native-like membrane mimetics, now enables near-atomic reconstructions of highly dynamic and previously intractable membrane proteins (Vinothkumar and Henderson, 2016). Critical to this progress has been the widespread adoption of antibodies–in particular, Fabs, scFvs, nanobodies, and engineered scaffolds–as conformational stabilizers, fiducial markers, rigidity enhancers, and molecular-weight augmenters (Hunte and Michel, 2002; Uysal et al., 2009; Steyaert and Kobilka, 2011; Wu et al., 2012) (Figure 1).

Simultaneously, artificial intelligence (AI) and machine-learning-based modeling have begun to influence protein-structure prediction, binder design, and structural interpretation (Jumper et al., 2021). These tools now complement experimental approaches by providing predictive insight into conformational landscapes, interface energetics, and scaffold engineering (Senior et al., 2020; Evans et al., 2021). In this review, we focus on antibody-enabled structural biology, detailing how antibodies and engineered scaffolds have expanded the tractable space of crystallography and cryo-EM, and how AI-driven computational methods augment these pipelines by assisting in binder discovery, structural modeling, energetic refinement, and *de novo* design. Our emphasis is experimental: AI is presented not as a replacement for structural biology, but as a toolkit that enhances and accelerates antibody-driven structural discovery.

## 2 Protein engineering and antibody technologies in structural biology

### 2.1 Engineering membrane protein targets for structural stability

Protein engineering has been pivotal in overcoming the intrinsic instability and conformational heterogeneity of membrane proteins, particularly G protein-coupled receptors (GPCRs), a superfamily that regulates diverse signaling pathways and represents nearly 50% of all current drug targets (Santos et al., 2017). Their structure determination was long hindered by conformational flexibility, poor solubility, and rapid denaturation outside lipid membranes. Here, “protein engineering” refers primarily to modifications of the target protein, such as stabilizing mutations, truncations, or fusion partners, rather than engineering of the antibody scaffold, which is discussed in Sections II.B and II.C.

The β_2_-adrenergic receptor (β_2_AR) became a landmark example in addressing these challenges. Although identified in the 1990s, its high-resolution structure was not solved until 2007, when Cherezov and colleagues used a combination of protein engineering and ligand stabilization (Cherezov et al., 2007; Rasmussen et al., 2007; Rosenbaum et al., 2007). Unlike rhodopsin, β_2_AR displays basal activity and a highly flexible intracellular loop 3 (IL3) that hindered crystallization. Stabilizing the inactive state with the inverse agonist carazolol, and either binding an IL3-specific Fab or replacing IL3 with T4 lysozyme (T4L), reduced conformational heterogeneity and produced crystals diffracting to 2.4 Å.

This work not only yielded the first non-rhodopsin GPCR structure but also established a general framework for dynamic membrane proteins. The T4L-fusion strategy, combined with lipidic cubic phase (LCP) crystallization, was rapidly adopted for other GPCRs, including adenosine A_2_A and μ-opioid receptors, with later adaptations introducing alternative fusion partners such as BRIL and PGS to further enhance stability and packing (Cherezov, 2011; Chun et al., 2012; Sounier et al., 2015).

Nanobodies have since emerged as transformative reagents in structural biology due to their small size, single-domain architecture, and exceptional conformational selectivity. Derived from camelid heavy-chain-only antibodies, they can access recessed or transient epitopes often inaccessible to conventional antibodies (Muyldermans, 2013). Their compact and rigid nature makes them ideal for capturing active or intermediate states of dynamic proteins such as GPCRs, ion channels, and transporters, and nanobody-assisted stabilization has become a cornerstone of membrane-protein structural biology (Pardon et al., 2014).

### 2.2 Antibody-derived fragments as structural chaperones

Antibodies have become indispensable in structural biology for conformationally dynamic and membrane-embedded proteins. By binding defined epitopes with high affinity and rigidity, full-length immunoglobulins and antibody fragments stabilize flexible regions, increase solvent-exposed surface area for lattice formation, and trap transient states, thereby enhancing crystallizability and enabling high-resolution structure determination by X-ray crystallography and cryo-EM. Among antibody-derived reagents, Fab fragments remain the most widely used format for cryo-electron microscopy, reflecting their favorable balance of molecular weight, rigidity, and ease of generation relative to other antibody formats. Early exemplars included Fab-assisted structures of cytochrome c oxidase and KcsA, which established antibodies as true structural scaffolds rather than merely biochemical probes (Uysal et al., 2009; Iwata et al., 1995; Zhou et al., 2001). A range of antibody-derived fragments and engineered binding scaffolds are now routinely used as structural chaperones in cryo-EM and crystallography, each offering distinct advantages and trade-offs in size, rigidity, and engineering complexity (Table 1).

**TABLE 1:** Comparison of commonly used antibody and antibody-derived formats in structural biology. Formats are summarized by approximate molecular size, key advantages and limitations, and typical applications in cryo-EM and crystallography.

Format	Typical size (kDa)	Key advantages	Key limitations	Common structural biology use
Fab	∼50	Well-established; high affinity; stabilizes conformational states	Larger size; flexible hinge region can limit resolution	Cryo-EM of membrane proteins, GPCRs; crystallographic chaperones
scFv	∼25–30	Smaller than fab; single chain, fusion-compatible	Often unstable; prone to aggregation	Limited structural use; fusion constructs
Nanobody (VHH)	∼12–15	Small; rigid; binds cryptic epitopes; effective state stabilizer	Limited mass contribution; affinity varies by target	Cryo-EM, crystallography, state stabilization
Megabody	∼100+	Increases particle mass; improves alignment and angular distribution	Requires fusion engineering and validation	Cryo-EM of small proteins
Legobody	Variable	Modular design; customizable geometry	Limited adoption; few validated examples	Cryo-EM scaffolding
DARPin	∼14–18	Rigid; high expression; tunable geometry	Epitope geometry constraints; reformatting less standardized than other antibodies	Cryo-EM scaffolds, cages
Scaffolded complexes	Variable	Symmetry amplification; signal enhancement for small targets	Assembly complexity; stoichiometry and heterogeneity risks	Cryo-EM of sub-50 kDa targets

Systematic deployment of Fabs, scFvs, and nanobodies transformed these isolated successes into a generalizable strategy for membrane-protein stabilization. By recognizing state-specific epitopes, antibody fragments “lock” targets into discrete conformations and reduce ensemble heterogeneity, improving phasing and packing for X-ray studies and boosting alignment fidelity and map interpretability for cryo-EM (Mukherjee et al., 2018; Yan et al., 2021; Erramilli et al., 2023). A canonical example is the nanobody Nb80, which stabilizes the active-state β_2_-adrenergic receptor by binding an intracellular epitope exposed upon activation, effectively locking the receptor in an active conformation (Rasmussen et al., 2011). More recently, this strategy has been extended to ion channels, as exemplified by nanobodies that allosterically bind the human α7 nicotinic acetylcholine receptor and stabilize a conductive state, revealing a distinct, pharmacologically-relevant potentiating mechanism (Prevost et al., 2023). Together, these examples illustrate how nanobodies in particular have extended this strategy to highly dynamic targets, broadening access to active-state GPCRs, ion channels, and transporters and enabling structures of signaling assemblies and intermediates that were previously inaccessible.

Beyond stabilization, antibody and antibody-like scaffolds act as fiducial markers in cryo-EM, providing distinctive extramembranous density that aids particle orientation and pushes the effective lower-mass boundary of single-particle analysis below ∼100 kDa, particularly when Fabs, nanobodies, or DARPins are used to increase contrast and break symmetry (Wentinck et al., 2022). A concise exemplar is KcsA, where antibody chaperoning materially advanced mechanistic resolution: monoclonal Fab fragments stabilized the tetrameric channel and improved crystal order, yielding a 2.0 Å Fab-KcsA complex that clarified ion coordination and hydration chemistry in the selectivity filter and connected pH-dependent gating to selectivity-filter dynamics (Zhou et al., 2001).

Collectively, antibody-based chaperones now bridge immunochemistry, molecular engineering, and high-resolution biophysics, stabilizing target states, adding fiducials for alignment, and reliably expanding the structural reach of both crystallography and cryo-EM across challenging membrane-protein systems.

### 2.3 Directed discovery *via* phage display and synthetic libraries

The rapid generation of conformation-specific antibody fragments has been transformed by phage-display technology and the emergence of synthetic antibody libraries (McCafferty et al., 1990; Hoogenboom, 2002). Phage display enables fully *in vitro* selection of high-affinity binders against purified targets, bypassing the need for immunization and permitting precise control over selection pressures for unstable or toxic membrane proteins (Paduch and Kossiakoff, 2017). Antibody variable domains displayed on filamentous bacteriophage undergo iterative rounds of biopanning, yielding clones that retain high specificity under detergent, amphipol, or nanodisc conditions that mimic native membrane environments (Dominik and Kossiakoff, 2015; Dominik et al., 2016).

The evolution of library design has further expanded this capability. Early semi-synthetic human Fab and scFv repertoires such as Griffin-1 and HuCAL demonstrated that large, fully *in vitro* libraries could yield functional binders, even to difficult membrane-associated targets (Dantas-Barbosa et al., 2012). Subsequent structure-guided libraries, including the minimalist Tyr/Ser-biased designs pioneered by Sidhu and colleagues, showed that restricted amino-acid alphabets can preserve broad specificity while improving developability and library quality (Fellouse et al., 2007). Modern phage and synthetic libraries are increasingly pre-filtered for developability, expression, and stability, reducing downstream attrition and improving performance under detergent, amphipol, or nanodisc conditions. In parallel, antibody and nanobody repertoires can be engineered to include elongated CDR loops that access recessed or occluded epitopes, such as ligand-binding pockets and clefts common in membrane proteins, enabling selection of binders that stabilize conformational states relevant to mechanism and pharmacology. More recently, a diverse ecosystem of synthetic and semisynthetic nanobody (VHH) libraries and alternative scaffold platforms such as DARPins has emerged, offering compact, stable, and conformationally selective binders well suited to GPCRs, ABC transporters, ion channels, and large multiprotein assemblies (Tamaskovic et al., 2012; McMahon et al., 2018). A prominent example is the nuclear pore complex, where phage-display-derived Fabs stabilized subcomplexes and enabled crystallization and high-resolution mapping of an assembly once considered structurally intractable (Stuwe et al., 2015).

These principles now extend directly into modern cryo-EM scaffolding systems. The NabFab architecture, pairing a nanobody bound to the target with a secondary Fab raised against the nanobody, creates rigid, asymmetric complexes that markedly improve alignment and signal-to-noise for proteins below ∼70 kDa (Bloch et al., 2021). Related constructs such as Megabodies (nanobody-scaffold fusions) and Legobodies (modular multivalent nanobody assemblies) apply the same design logic, increasing effective molecular weight and geometric asymmetry to enhance particle orientation and image alignment (Uchański et al., 2021; Wu and Rapoport, 2021). Collectively, these modular scaffolds have pushed cryo-EM into regimes previously inaccessible, enabling high-resolution reconstruction of small and flexible membrane proteins and enzymes.

Phage-display and synthetic-library technologies have therefore transformed antibody discovery from an empirical art into a rational, high-throughput, and tunable discipline. The resulting reagents function not only as structural stabilizers but also as mechanistic probes capable of defining conformational equilibria, ligand interactions, and allosteric transitions with molecular precision. When integrated with computational modeling and AI-based sequence optimization, these approaches form a unified experimental-computational framework for structure-guided drug discovery, bridging biophysics, binder engineering, and therapeutic innovation.

## 3 Antibody-fragment applications in structural biology

### 3.1 Case studies demonstrating structural impact

Antibody fragments, including nanobodies, Fabs, and engineered scaffolds such as DARPins, have significantly expanded the structural reach of cryo-EM and crystallography. By stabilizing transient conformations, increasing molecular weight, and imposing rigidity, these reagents have opened access to membrane proteins and small targets that had long resisted high-resolution study. Several recent examples illustrate the breadth of their impact.

Nanobodies have been especially effective at capturing conformationally elusive transporters. Geldreich and colleagues applied a high-affinity nanobody to the Lactococcus lactis ABC transporter EfrCD, a dynamic efflux pump implicated in multidrug resistance (Meier et al., 2023). Co-expression of the nanobody locked EfrCD in a well-defined inward-facing conformation, increasing the mass and rigidity of the complex and enabling a 4.25 Å cryo-EM reconstruction. The resulting structure revealed the organization of the inward-facing cavity, the orientation of the nucleotide-binding domains, and the peristaltic motions that drive substrate extrusion, underscoring the power of nanobody-assisted state trapping for mechanistic analysis of transporter cycles.

Fabs can play a related but distinct role by modulating receptor activity. Saotome et al. used the therapeutic antibody REGN7663 to solve the structure of the CXCR4–CXCL12 complex, a GPCR–chemokine system with critical roles in immune trafficking, metastasis, and viral entry (Saotome et al., 2025). The CDR-H3 loop of the Fab penetrated the CXCR4 orthosteric pocket and acted as an inverse agonist, reshaping the receptor’s conformational ensemble. Cryo-EM analysis revealed trimeric and tetrameric states of CXCR4, pointing to higher-order organizational principles not fully appreciated from previous structures and illustrating how Fabs can both stabilize receptors and reveal allosteric mechanisms relevant to pharmacology.

Engineered scaffolds such as DARPins have emerged as powerful tools for visualizing small proteins below the practical mass threshold of single-particle cryo-EM. Castells-Graells and colleagues developed a modular DARPin–protein cage system that symmetrically decorates a rigid protein cage with target-binding DARPins, thereby amplifying the apparent molecular weight and enforcing a well-defined geometric arrangement of the bound targets (Castells-Graells et al., 2023). Using this approach, they achieved near-atomic (∼3 Å) reconstructions of small proteins, including GFP and inhibitor-bound KRAS (G12C), resolving features such as the GFP chromophore and small-molecule binding modes. Crucially, the high symmetry and rigidity of the DARPin cage enable symmetry-based averaging during image processing, substantially improving alignment, signal-to-noise, and angular sampling. This strategy overcomes preferred-orientation and low-contrast limitations that have historically precluded atomic-resolution cryo-EM analysis of proteins below ∼50 kDa.

### 3.2 Integration and broader implications

Collectively, these studies exemplify how engineered antibody fragments, whether derived from immune repertoires or synthetic scaffolds, have expanded the frontiers of structural biology. Nanobodies offer conformational specificity and small size ideal for capturing transient intermediates. Fabs combine mass and geometric precision, enabling orientation control and direct insight into ligand modulation. DARPins provide thermostability and modularity, serving as designable scaffolds that extend cryo-EM to the sub-50 kDa regime.

Together, these technologies now constitute a cohesive toolkit for addressing the intrinsic limitations of membrane-protein and small-protein structural biology. Their integration with modern cryo-EM workflows and computational modeling has redefined the achievable scope of structure determination, transforming previously intractable targets into systems amenable to atomic-level analysis. As AI-driven modeling and *in silico* binder design continue to evolve, the convergence of antibody engineering, scaffold development, and cryo-EM promises an unprecedented era of precision structural biology.

## 4 Molecular-weight augmentation and symmetric scaffolding in Cryo-EM

### 4.1 The challenge of small-protein reconstruction

Despite transformative advances in direct-electron detection and image processing, single-particle cryo-EM (SPA) continues to face a persistent size barrier. Small proteins (<100 kDa) suffer from low signal-to-noise ratios and limited morphological features for accurate particle alignment. Although nearly 75% of the human proteome is smaller than 50 kDa, less than 3% of cryo-EM structures resolved at better than 4 Å fall within this range (Herzik et al., 2019). To bridge this gap, researchers have developed molecular-weight augmentation and rigidity-enhancement strategies that increase effective particle size and orientation diversity while maintaining native conformation.

### 4.2 Antibody-based molecular-weight augmentation

Antibody fragments, particularly Fabs and nanobodies, remain the most versatile augmentation agents because they combine mass increase, conformational stabilization, and symmetry breaking in a single molecular tool. Their application extends beyond passive binding: through structural engineering, antibody fragments have been transformed into modular augmentation systems optimized for cryo-EM.

The NabFab approach exemplifies this principle. In this strategy, a Fab is raised not against the target protein directly but against a universal nanobody framework (Bloch et al., 2021). The nanobody recognizes and locks the target protein, while the secondary Fab binds the nanobody itself, effectively doubling the complex’s molecular weight and providing multiple fiducial markers for particle alignment This two-component system has enabled the visualization of small membrane proteins, achieving near-atomic reconstructions of sub-70 kDa complexes.

Building on this concept, Megabodies are created by genetically fusing nanobodies to rigid monomeric scaffolds such as HopQ or small enzyme domains, thereby increasing apparent molecular weight and introducing geometric asymmetry. This method proved particularly successful for the 62-kDa hedgehog acyltransferase (HHAT) enzyme, whose structure was resolved at 2.7 Å^39^. Legobodies, multivalent nanobody assemblies tethered by flexible linkers, offer modular combinations of epitopes and several fiducials per complex, facilitating sub-3 Å reconstructions of small viral proteins, including the SARS-CoV-2 receptor-binding domain (RBD) (Wu and Rapoport, 2021).

Collectively, these antibody-based augmentation systems demonstrate how structural biologists have repurposed immunochemical diversity into a rational framework for cryo-EM signal amplification, expanding the method’s lower mass limit by nearly an order of magnitude.

### 4.3 Fusion-protein strategies for rigidity and contrast

While antibody complexes augment molecular mass non-covalently, fusion-protein strategies covalently attach large, rigid domains to the target. These methods improve SNR by increasing overall particle size and imposing structural rigidity, thereby mitigating the blurring effects of conformational flexibility. Fusion partners such as BRIL, T4 lysozyme (T4L), and the pyruvate-phosphate dikinase regulatory domain (PGS) have been repeatedly deployed to stabilize transmembrane helices and loop regions (Chun et al., 2012). These fusions not only enhance expression and stability but also create anchor points for high-affinity Fabs, combining fusion-based rigidity with antibody-based contrast (Mukherjee et al., 2020).

Recent innovations extend this principle further. The NanoBiT system employs split luciferase fragments fused to GPCR termini, providing a luminescent reporter and an additional rigid interface useful for orientation control in cryo-EM (Duan et al., 2020; Reyes-Alcaraz et al., 2022). Similarly, epitope fusions recognized by engineered antibodies, such as the BRIL-binding Fab used for the Frizzled five receptor, and engineered intracellular loop motifs, such as the κ-opioid receptor ICL3 recognized by specific nanobodies, allow consistent orientation across diverse targets while maintaining physiological relevance (Tsutsumi et al., 2020).

Fusion-protein augmentation thus represents a balance between mass increase and conformational integrity, yielding constructs optimized for both structural stability and biological fidelity.

### 4.4 Symmetric scaffold architectures for signal enhancement in Cryo-EM

A complementary route to molecular-weight augmentation lies in symmetry-based scaffolding, which attaches small proteins to pre-organized multimeric assemblies that amplify total particle mass and orientation diversity. These scaffolds enhance effective SNR and mitigate preferred-orientation artifacts, persistent challenges in high-resolution imaging of small or symmetric proteins.

Apoferritin, a naturally occurring 24-subunit nanocage exhibiting octahedral symmetry, has become one of the most effective scaffolds for this purpose. Genetic fusion of target peptides or domains to apoferritin subunits enables controlled display of small proteins at defined vertices, markedly improving both contrast and angular sampling. Early “nanohedra” designs showed that rigid α-helical linkers could drive assembly of symmetric cages and filaments, providing a conceptual basis for later nanocage architectures such as ferritin and computationally engineered assemblies (Padilla et al., 2001).

Building on these foundations, apoferritin-based scaffolds have been adapted to present small proteins or domains in precisely defined orientations, allowing visualization of targets well below the typical cryo-EM detection threshold. For example, Lu et al. created a DARPin–apoferritin hybrid scaffold, an ∼1 MDa assembly that enabled near-atomic reconstructions of small model proteins such as GFP and maltose-binding protein (Lu et al., 2025). Castells-Graells et al. demonstrated that DARPin-cage scaffolds can achieve ∼3 Å resolution for sub-25 kDa therapeutic targets, including the KRAS G12C mutant bound to AMG510 (Castells-Graells et al., 2023). In both cases, *in silico* modeling was used to pre-optimize interface geometry and rigidity, illustrating the convergence of computational protein design, symmetry modeling, and cryo-EM methodology.

These symmetric architectures mark a turning point in structural biology, transforming molecular-weight augmentation from *ad hoc* tagging into predictive, design-driven engineering and paving the way for AI-driven scaffold discovery.

### 4.5 Computational design and experimental validation of protein scaffolds

The design of molecular-weight augmentation constructs has shifted from empirical trial-and-error toward predictive, computation-guided engineering (Jumper et al., 2021; Watson et al., 2023; Krishna et al., 2024). Deep-learning–based structure prediction and generative protein-design tools now propose scaffolds that can be pre-screened *in silico* for rigidity, interface geometry, and orientation distributions before experimental testing.

Physics-based refinement complements these models by assessing dynamic and energetic feasibility. Molecular-dynamics simulations quantify linker flexibility and interface stability under cryo-EM–like conditions, while macromolecular modeling suites estimate hydrogen-bond networks, van der Waals complementarity, and packing (Alford et al., 2017; Hollingsworth and Dror, 2018). Together, these calculations guide linker optimization and scaffold rigidification, increasing the likelihood that designed constructs will perform as intended.

Importantly, current *in silico* scaffold- and binder-design pipelines still require experimental validation prior to cryo-electron microscopy. Designed proteins are typically triaged by expression and biophysical quality (e.g., size-exclusion chromatography behavior and thermostability), followed by binding assays such as surface plasmon resonance (SPR), biolayer interferometry (BLI), ELISA, or pull-down experiments before committing to negative-stain EM or cryo-EM, which remain comparatively low throughput (Bennett et al., 2023). While this validation step represents a practical bottleneck, recent advances are beginning to reduce the experimental screening burden; for example, BindCraft reports cases in which only a small number of designed candidates need to be tested to identify a functional binder, foreshadowing more efficient closed-loop design-test cycles (Bennett et al., 2025). The effectiveness of these cycles depends critically on the availability of high-quality experimental data describing expression, stability, solubility, and binding behavior, which provide the ground truth needed to train, benchmark, and refine computational models (Watson et al., 2023; Fox et al., 2025).

Taken together, AI prediction and empirical validation now form an increasingly efficient closed-loop design framework, in which deep-learning models generate candidate constructs, physics-based tools evaluate mechanical and energetic plausibility, and cryo-EM confirms structural reality. This iterative framework transforms augmentation into a predictive engineering discipline, capable of tailoring symmetry, stability, and mass with near-atomic foresight.

### 4.6 Outlook

The convergence of antibody engineering, symmetric scaffolding, and computational design is rapidly transforming cryo-EM from an empirical pursuit into a predictive design discipline. What once required years of iterative optimization can now be approached through integrated computational–experimental cycles that couple molecular-weight augmentation with algorithmic foresight. As these pipelines mature, high-resolution reconstructions of proteins below 50 kDa should become increasingly routine, positioning cryo-EM, augmented by rationally designed binders and scaffolds, as a central engine for structure-guided discovery.

## 5 Artificial intelligence in structural biology and antibody design

The integration of AI and machine learning has begun to shift structural biology from a predominantly empirical endeavor toward a more predictive, design-driven discipline. Deep-learning architectures now interpret protein sequences and predict three-dimensional structures with remarkable accuracy, complementing approaches such as cryo-EM and X-ray crystallography (Jumper et al., 2021; Watson et al., 2023). When incorporated into antibody-engineering workflows, these computational systems accelerate multiple stages of design, from epitope mapping and paratope identification to energetic optimization, creating an increasingly tight loop between *in silico* prediction and experimental validation.

### 5.1 Predictive modeling and structural refinement

Deep-learning frameworks such as AlphaFold2 and RoseTTAFold, together with transformer-based language models, learn contextual residue relationships from large sequence datasets and effectively encode the “grammar” of protein folding (Jumper et al., 2021; Baek et al., 2021). When adapted to antibody repertoires, these models provide priors on CDR conformations, overall folding stability, and candidate paratope–epitope interfaces, thereby accelerating early stages of antibody and nanobody discovery (Ruffolo et al., 2023).

Neural predictions are increasingly coupled to physics-based refinement. Molecular dynamics engines enable quantitative assessment of conformational stability, hydrogen-bond persistence, and solvent exposure over extended timescales, while macromolecular modeling suites perform flexible-backbone refinement, side-chain repacking, and energy minimization to improve local geometry and interface complementarity. Together, these hybrid workflows combine AI-predicted global folds with atomistic molecular mechanics to refine flexible loops and optimize antibody–antigen interfaces before experimental screening.

### 5.2 AI-enhanced antibody design pipelines

Beyond structure prediction, AI now contributes directly to the *de novo* design and optimization of antibody sequences (Watson et al., 2023). Generative and structure-conditioned models, including diffusion-based backbone design frameworks, sequence-design networks such as ProteinMPNN, and antibody-focused predictors like IgFold, enable targeted engineering of antibodies and nanobodies (Ruffolo et al., 2023; Dauparas et al., 2022). Diffusion models propose structural scaffolds and binding geometries, while sequence-design algorithms assign compatible amino-acid sequences. Antibody-specific predictors rapidly assess CDR configuration, structural plausibility, and developability, allowing focused mutational exploration around promising paratopes.

Recent workflows integrate these predictive tools with atomistic refinement. As highlighted in, for example, Reddy et al., AI-based structure prediction can be combined with molecular-dynamics simulations and free-energy methods to refine candidate interfaces and evaluate mutational effects (Reddy et al., 2024). In such pipelines, AI-generated variants are assessed for changes in binding free energies, conformational entropy, and stability in near-physiological environments, enabling triage of large numbers of variants before laboratory synthesis.

Increasingly, these methods are integrated into unified computational environments that link neural-network predictions with physics-based evaluation. Molecular-dynamics simulations probe local flexibility, hydration, and interface stability, while alchemical free-energy calculations estimate relative changes in affinity across mutational landscapes (Zheng et al., 2025). Complementary interface-analysis tools quantify hydrogen bonding, electrostatic complementarity, and desolvation, informing rational CDR optimization. Visualization frameworks that combine cryo-EM densities, AI-predicted models, and MD trajectories enable direct comparison between predicted and experimental structures, completing a closed loop between computation and experiment (Wang et al., 2024).

Collectively, these AI-driven platforms are reshaping antibody engineering. Rather than serving as passive supplements, neural and physics-based models now function as active design engines that propose, evaluate, and iteratively refine molecular hypotheses prior to laboratory execution (Pac et al., 2025). This convergence of deep learning, statistical modeling, and molecular simulation marks a transition toward next-generation structure-guided discovery, one in which *in silico* reasoning directly informs experimental design and significantly narrows the search space for high-affinity, developable antibody candidates (Bennett et al., 2025).

## 6 Future perspectives and therapeutic implications

### 6.1 Toward a unified experimental–computational framework

Structural biology is entering a phase in which experimental precision and computational prediction operate as components of a single, iterative system. Antibody-assisted crystallography, NMR spectroscopy, and integrative structure determination are now complemented by deep-learning-based inference that can propose conformational states, alternative assemblies, and dynamic transitions with high confidence. As structural datasets continue to grow, adaptive AI frameworks will progressively refine themselves, incorporating new diffraction, cryo-EM, and NMR data and updating their internal representations of folds and interfaces.

For antibody and binder discovery, this convergence enables rapid identification of conformationally selective ligands rather than binders defined solely by affinity. When phage-display libraries, AI-driven paratope prediction, and physics-based scoring are combined, antibodies can be designed to stabilize specific target states and tailored to deliver therapeutic impact. The field is thus moving from a paradigm in which structural biology primarily observes molecules to one in which it increasingly engineers them.

### 6.2 Expanding therapeutic reach

Antibody-guided structural elucidation has broad therapeutic implications. High-resolution structures of active, inactive, and allosterically modulated conformations of key targets, including receptor tyrosine kinases, immune checkpoint proteins, GPCRs, and ion channels, now underpin rational design of monoclonal antibodies, small molecules, and hybrid biologics (Congreve et al., 2020). Nanobody- and Fab-stabilized complexes have informed the development of bispecific antibodies and engineered chimeric antigen receptor T-cell (CAR-T) constructs, demonstrating a direct link between structural insight and advanced immunotherapies (Almagro et al., 2018; Fer et al., 2023).

In parallel, antibody scaffolds are being deployed as modular imaging and diagnostic tools capable of reporting on drug–target engagement *in situ* (Li et al., 2023). Coupled with MD- and AI-based predictions, structural data can anticipate resistance mutations, allosteric drift, and binding-mode switching–factors central to therapeutic durability in oncology, neurology, and infectious disease.

## 6.3 Challenges and opportunities

Despite rapid progress, several key challenges remain. Stabilizing antibody–antigen complexes in native or native-like membrane environments still demands improved display technologies, more sophisticated sample-preparation pipelines, and computational pre-screening for thermodynamic robustness. Integrating heterogeneous datasets from crystallography, cryo-EM, NMR, and simulation requires cross-modality alignment methods capable of distinguishing genuine conformational heterogeneity from noise, and reproducibility will benefit from standardized deposition and annotation protocols for AI-augmented structural models. A complementary opportunity, depending on the structural question being addressed, is the generation of soluble membrane-protein analogues that preserve key architectural and functional features while circumventing native sample-preparation constraints, as demonstrated by recent *de novo* designed mimetics (Goverde et al., 2024).

Emerging technologies provide unprecedented opportunity. Advances in time-resolved crystallography, integrative structural modelling, and native-environment NMR are enabling the capture of molecular motion across biologically relevant timescales. When combined with AI-guided conformational mapping and free-energy-landscape computation, these developments will illuminate transient intermediates in folding, catalysis and signaling, ushering in a holistic, time-resolved view of structure and function.

## 7 Conclusion

Antibodies and their engineered derivatives have evolved from purely immunological reagents into foundational tools of structural biology. Their ability to stabilize, orient, and define target conformations has extended the reach of crystallography, cryo-EM, NMR, and hybrid methods from complexes once deemed intractable to systems that now serve as routine platforms for mechanism and design. In parallel, AI and physics-based modeling have begun to transform how these structures are predicted, refined, and exploited for therapeutic purposes, enabling *in silico* exploration of mutational space and binding energetics that would be impractical to probe experimentally at scale.

The convergence of experimental observation and computational prediction marks a turning point: structural biology is increasingly predictive and generative rather than solely descriptive. It now operates as a dynamic discipline in which algorithms and experiments co-evolve, allowing simulation, validation, and targeted manipulation of molecular systems before they are synthesized or expressed. Looking ahead, the fusion of antibody engineering, integrative structural methods, and adaptive AI pipelines will enable researchers to design, test, and refine biological macromolecules with unprecedented precision, ushering in an era in which structural information not only explains function but also anticipates and shapes it.
